# Supplementary material for: Subnational tailoring of malaria interventions for strategic planning and prioritization: Experience and perspectives of five malaria programs
Source: PLOS Glob Public Health. 2025 May 28;5(5):e0003811. doi: 10.1371/journal.pgph.0003811 (PMC12118815; doi:10.1371/journal.pgph.0003811)
Supplement: S1 File — (DOCX) [file pgph.0003811.s001.docx]

**Informed Consent Form**: **Qualitative assessment of NMCPs’ perceptions of the stratification and sub-national tailoring of interventions analytical processes**

**Principal Investigators:** Abdisalan Noor/Jaline Gerardin

**Organizations:** World Health Organization/Northwestern University

**Sponsor:** Bill and Melinda Gates Foundation

**Project Name:** Qualitative assessment of NMCPs’ perceptions of the stratification and sub-national tailoring of interventions analytical processes

**Introduction**

My name is [Letitia Onyango/Ghislaine Ouédraogo-Ametchie] and I am a researcher at Northwestern University. We are conducting a study of NMCPs perspectives of sub-national tailoring of intervention activities implemented with WHO and analysis partners. I am going to provide some background information about the study so that you understand what your participation entails. Your participation in this study is entirely your choice, and you do not have to provide your decision at this moment. This consent may contain some terms you are not familiar with. If that happens, please stop me so that I can take some time to explain. If you have questions after we have gone through this consent together, you can ask me or another researcher before you make your decision.

**Purpose of the research**

You may recall that recently, your team organized and analysed all available data to guide stratification of the country and targeting of interventions in close collaboration with WHO. Your team organized and analysed all available data to guide stratification of the country and targeting of interventions, and external partners including geospatial and mathematical modellers were recruited to help with some analyses. We would like to learn what NMCP staff thought about this process. This includes what NMCP staff believe went well, what did not go as well, and recommendations for improving activities like this in the future. We would like to learn your experience with the overall process, and learn how to improve analysis support for NMCPs in the future.

**Type of Research Intervention**

This study will involve your participation in an in-depth interview that will take about one hour and a half, and a short survey that will take no more than ten minutes to complete.

**Participant Selection**

You are being invited to take part in this research because of your participation in these activities, and your experience with your NMCP. We believe you can contribute to our understanding of how analysis can support NMCPs to strategize interventions.

**Voluntary Participation**

Your participation in this study is entirely voluntary. This means that it is your choice to participate, or not participate. There are no consequences for choosing not to participate. Your participation will not impact your NMCP’s current or future partnerships with WHO. If you do choose to participate, you can choose to stop participating at any time.

**Procedures**

We would like to ask you some questions about your experience with the subnational tailoring analysis/stratification process. If you accept to participate, we will conduct an interview with you, followed by a very brief survey.

The interview will be a virtual discussion between you and our interviewer. Before the interview, you will be asked to provide your availability for the next two to three weeks. Once you provide your availability, I or another interviewer will send you a Zoom link to conduct the interview. Only the interviewer and a note-taker will be present for the interview. You may choose to be on or off camera for the duration of the interview. If you do not wish to answer any of the questions during the interview, you may say so and the interviewer will move on to the next question. The information recorded is confidential, and no one else except the research team will have access to the information documented during your interview. The entire interview will be audio-recorded, but no-one will be identified by name on the recording. These recordings will be transcribed. Your name will not appear on the transcript. Both the audio recording and transcript will be stored on a password-protected database, only accessible by the researchers associated with this study. The interview audio will be destroyed fifteen weeks after your interview. However, you can request a copy of your transcript at any time.

After the interview, you will receive a link to a survey that you will fill out on your own. If you do not wish to answer any of the questions in the survey, you can skip them and move on to the next question. Your name will not appear on the survey, and all of your responses will be confidential. Your name will not appear anywhere on the survey forms, only a number will be used to link your survey to your interview. No one outside the research team will have access to your survey.

Following the interview, you may be contacted again to clarify any information provided, or to follow up on some of the findings emerged during analysis.

**Duration**

For this study, you will be interviewed one time, and take the survey one time. The interview is expected to last one hour to ninety minutes, and the survey is expected to last no more than ten minutes.

**Risks**

There is a risk that you may share some personal or confidential information by chance, or that you may feel uncomfortable talking about some of the topics. However, we do not wish for this to happen. You do not have to answer any question or take part in the discussion/interview/survey if you feel the question(s) are too personal, if talking about them makes you uncomfortable, or if you prefer not to answer.

**Benefits**

There are no direct benefits to you, but your participation will likely help us understand how to improve analysis support for NMCPs in the future.

**Reimbursements**

You will not receive any incentive to participate in this research.

**Confidentiality**

We will not be sharing information about you to anyone outside of the research team. The information that we collect from this research project will be kept private. Any information about you will have a number on it instead of your name. Only the researchers will know what your number is and we will store this information in password-protected folders.

We will not ask you about child abuse, but if you tell us about child abuse or neglect, we may be required by law or university policy to report to authorities.

**Sharing the Results**

Nothing you share during your interview will be shared outside the research team, and nothing will be attributed to you by name. The knowledge we get from this research will later be shared with you and your NMCP before it is made available to anyone else. The findings from this research may also be used to produce academic manuscripts. We will not use your name for these manuscripts, but we may indicate that we interviewed someone from your country’s NMCP. We will seek your permission before using any quotes. We will hold small meetings with your NMCP and other NMCPs to discuss the results of this research. Following these meetings, we will publish the results so that others who are interested can learn from this research. You can choose whether or not you want any of your interview appearing in future publications. Your name will not be tied to any data in publications at any time.

**Right to Refuse or Withdraw**

You do not have to take part in this research if you do not wish to do so. Choosing to participate will have no effect on your position, your NMCP’s current or future partnerships, or your relationship with WHO or with analysis partners. You may choose to stop participating at any time without any consequences. At the end of the interview, I will give you an opportunity to let me know if there are parts of the interview you would prefer removed.

**Who to Contact**

This research has been reviewed and approved by an Institutional Review Board (“IRB”) – an IRB is a committee that protects the rights of people who participate in research studies. You may contact the IRB by phone at +1 (312) 503-9338 or by email at [irb@northwestern.edu](mailto:irboffice@organization.org) if:

- Your questions, concerns, or complaints are not being answered by the research team.
- You cannot reach the research team.
- You want to talk to someone besides the research team.
- You have questions about your rights as a research participant.
- You want to get information or provide input about this research.

You can ask me any questions about any part of the research study if you wish. Do you have any questions?

**Consent Form**

Please indicate your consent to participate in this study using the following link. As a reminder, you will have an opportunity to ask questions on the day of your interview. You may also change your mind about participation at any time, without consequence.

Consent or decline to participate (link to online consent form)
