# Supplementary material for: Subnational tailoring of malaria interventions for strategic planning and prioritization: Experience and perspectives of five malaria programs
Source: PLOS Glob Public Health. 2025 May 28;5(5):e0003811. doi: 10.1371/journal.pgph.0003811 (PMC12118815; doi:10.1371/journal.pgph.0003811)
Supplement: S2 File — (DOCX) [file pgph.0003811.s002.docx]

**Interview Guide: Qualitative assessment of NMCPs’ perceptions of the stratification and sub-national tailoring of interventions analytical processes**

Thank you for agreeing to your participation and taking the time to speak with me. I will commence with asking you a few questions regarding your experience with stratification and sub-national tailoring of interventions to inform national strategic plans at your NMCP. You may recall that your team recently organized and analysed all available data to guide stratification of the country and targeting of interventions, in close collaboration with the World Health Organization. Mathematical modellers were recruited to develop models to predict the impact of strategic and prioritized plans.

These questions will focus on your experiences, opinions, and recommendations regarding the analysis for stratification and sub-national tailoring of interventions. We would like to hear from you to understand your experience with stratification and sub-national tailoring, and identify ways to improve analysis support for NMCPs in the future.

Please note your participations in the collection of information of your experience remains voluntary. You may stop participating at any time or skip any questions that may make you uncomfortable.

Please remember there is no right or wrong answer. We would like you to speak freely, and share all of your experiences, positive or negative. In our publications, however, we may select a quote from this interview to illustrate a finding. Your name or position will never appear in these publications, but we will seek your permission before using any quotes in any presentations or publications. If you do allow us to use a quote from this interview, we will use a pseudonym. At the end of the interview, I will ask you again if you would still like to remain with the option you selected. You can change your mind any time.We will be recording this conversation in order to transcribe the audio recording. Your name will not appear on the transcript or the audio recording.

Do you have any questions before we proceed?

**NMCP’s recall of the overall SNT experience, its process and perceived outcomes (15min)**

1. Based on your recollection, what did the stratification and sub-national tailoring exercise that took place in partnership with WHO and analysis partners consist of?
   1. Can you describe the **overall process** and what it led to?
   2. What was your role in this process?
2. What were your **expectations** of this activity?
   1. Who were the **key partners** for this activity? What were their roles?
3. Was your NMCP motivated to engage with this activity? Why or why not?

**NMCP decision making processes related to NMSP development [NMCP manager, surveillance/M&E staff]**

1. Please describe the strategic plan development process at your NMCP. [probe: initiation, meetings, revisions]
   1. What is your **role** in this process?
   2. At what **period** does your NMCP conduct strategic planning?
   3. How **long** does each strategic plan last?
   4. Are there other key **players**? Who are they?
2. In NMSP cycles before using the stratification and sub-national tailoring approach, **how** did your NMCP develop strategic targets? [probe: WHO targets, MoH targets, evidence-based, previous experience]
3. In NMSP cycles before using the stratification and sub-national tailoring approach, did your NMCP use any **type of data** or analysis to develop strategic plans?
4. In NMSP cycles before using the stratification and sub-national tailoring approach, **which** **data** did you use to develop strategic plans? [probe: routine malaria case data, DHS data, other country level data, intervention data]
5. Did your country use any modeled data sources such as under-5 mortality or risk maps?
6. How was the data **analyzed**, and by whom?
7. In NMSP cycles before using the stratification and sub-national tailoring approach, did your NMCP consider regional or other subnational differences in developing strategic plans?
   1. [if yes] How were **regions/districts** differentiated or stratified?
   2. [if yes] could you elaborate on the **analysis** and results available, and the person/institution that conducted them?
   3. [if yes] how did it differ from the analytical support provided as part of stratification and sub-national tailoring?
   4. Did the previous NMSP consider any sort of sub-national tailoring of interventions? If so, could you explain in which instances were interventions targeted and by whom were they conducted?
   5. How did the NMSP development process change as a result of SNT? Which aspects changed the most?
   6. Were **mathematical models** ever used for malaria before in your country to inform the impact of your strategic plans? If so, could you explain in which instances were mathematical models used and by whom were they conducted?

**Reported use-cases of the results from the SNT analysis**

1. How did your NMCP primarily **use the results** from SNT analysis?
   1. Did these results **change any internal processes**? In what way?
   2. Which processes **benefitted** the most from SNT analysis?
2. Which internal factors enabled the use of analysis products? *[probe: NMCP enthusiasm for products, impact of SNT analysis on internal efficiencies]*
3. Which external factors enabled the use of analysis products? *[probe: competing external priorities, pressure from implementers]*
4. Do you believe outputs provided **feasible recommendations** for your NMCP? Why or Why not?
5. Do you think your NMCP has the **capacity to implement recommendations** from SNT analysis? Why or why not?
   1. [if no] What kind of **support** would your NMCP need to be able to implement recommendations from SNT analysis?
6. Mathematical modellers provided impact projections for strategic and prioritized plans derived from the stratification process. What kind of discussions did your NMCP have about the **use of mathematical models** and the resultant projections, if any? [probe: accuracy, usefulness, level of understanding]
   1. In what ways did the scenario results influenced your **Global Fund requests** and **national strategic plans**?

**Sense of ownership, leadership and accountability of the process and the results obtained**

1. Do you feel like you were **in charge** of the overall stratification and SNT analysis process? If not, who was?
   1. Which of the SNT analysis process [data analysis, stratification, intervention targeting, consensus building, funding request preparations] were you **involved in**? Which were you **in charge** of?
   2. Were there any parts of the process you felt **less included in**, or less in control of? What were they?
   3. Were there any instances where you felt an important decision was made **without** **your** **input**?
   4. [if examples provided] Did you address this with your **colleagues**? How about **analysis** **partners**?
2. Do you feel **responsible** for the **quality** and **timeliness** of the SNT analysis products? If not, who was?
3. Did you feel **invested** in the analysis process? Why or why not?

**Perceived capacity to improve or influence the analysis through local knowledge and technical expertise**

1. Overall, did you feel that analysis partners **valued your expertise**? [probe: local context, intervention knowledge]
   1. Did analysis partners actively **seek your inputs**? How so?
   2. How did your NMCP manage **disagreements** with analysis partners?
   3. Were there **specific elements** that caused more disagreements than others? What were they?
   4. How can analysis partners **better incorporate your expertise**?
2. Were there any aspects of this process that were **not clear** to you? If so, which ones?
   1. What did you or your **colleagues** do if something was not clear?
   2. Did you feel **comfortable** asking questions when something was not clear?
3. Were there any instances where you thought a map, modelled scenario, or other output was **inaccurate**? [if yes]
   1. How did your NMCP **manage** situations like this?
   2. Did you feel that you could **communicate** your perspectives to analysis partners? Were analysis partners **receptive** to this feedback?
   3. Were you satisfied with your ability to provide feedback on maps and models? Why or why not?

**Lessons learned, and suggestions on what to do differently in the future to improve acceptability and use of results**

1. In your opinion, what are the most important lessons learned as a result of the stratification and subnational tailoring analysis process?
2. What would you do differently in the future to improve acceptability and use of results?
3. In the future, how can analysis partners improve the overall quality of analysis products and the analysis process? *[probe: time spent providing explanations, time allocated for discussions, format of presentations, incorporation of local data, incorporation of local knowledge]*
4. In the future, how can analysis partners make stratification maps, mathematical modelling results and other outputs more useful for your NMCP’s priorities?

**Recommendations on ways to communicate and engage more effectively with WHO and analysis partners**

1. Most analysis results were shared directly through PowerPoints and long presentations. What did you think of this format?
   1. Is there anything you would change about the format of presentation results? [probe: clarity, presentation duration, language]
2. Is your NMCP invested in using analysis of this kind in the future? Why do you say this?
3. In your opinion, does your NMCP have the capacity to independently carry out any of the analysis activities we’ve discussed?
   1. Which skills are needed to improve your NMCP’s capacity to carry out analysis activities without external assistance?
   2. What type of support would be help your NMCP improve its capacity?
4. Do you believe your NMCP will continue to use analysis products? (stratification maps, mathematical models, etc) Why or why not? If your NMCP were to do this again, what would you differently? Why do you say this?
