# Supplementary material for: Subnational tailoring of malaria interventions for strategic planning and prioritization: Experience and perspectives of five malaria programs
Source: PLOS Glob Public Health. 2025 May 28;5(5):e0003811. doi: 10.1371/journal.pgph.0003811 (PMC12118815; doi:10.1371/journal.pgph.0003811)
Supplement: Attachment — Submitted filename: Review_PGPH-D-24-02161_Rajvanshi (1).docx [file pgph.0003811.s004.docx]

**Subnational Tailoring of Malaria Interventions for Strategic Planning and Prioritization: Experience and Perspectives of Five Malaria Programs (PGPH-D-24-02161)**

**Review by Dr Harsh Rajvanshi**

The manuscript presents a qualitative study exploring the perspectives of National Malaria Programs (NMPs) on the use of Subnational Tailoring (SNT) for malaria strategic planning and prioritisation. While the study is valuable in documenting experiences across five countries, several areas require improvement to enhance clarity, strengthen the analytical depth, and improve the overall presentation.

**Major Comments**

1. Conceptual Clarity and Rationale for SNT Approach

- The manuscript describes SNT as a novel approach, but does not clearly differentiate it from existing malaria stratification methods used in endemic countries.
  - How does SNT differ from traditional malaria stratification approaches used by WHO or NMCPs?
  - What are the specific advantages of SNT over conventional methods in terms of decision-making?
  - Was there a preceding malaria stratification framework in these countries before adopting SNT?
  - Suggested Revision: Provide a clearer theoretical grounding for SNT and its distinct value-add.

2. Overuse of WHO-Centric Perspective

- The WHO's role in SNT implementation is strongly emphasized, but the manuscript lacks a critical discussion on:
  - How NMPs perceived WHO’s role—was it more of a technical enabler or a decision-making authority?
  - Did WHO’s involvement introduce limitations, such as prescriptive frameworks that may not fully align with country-specific needs?
  - Suggested Addition: Provide a balanced discussion on both facilitating and limiting aspects of WHO’s involvement.

3. Participant Selection and Generalizability

- The study relies on interviews with 12 NMP representatives from five countries. However:
  - Why were only NMP representatives included? Were perspectives from other stakeholders (e.g., funders, health workers, community leaders) considered?
  - Suggested Revision: Acknowledge the limited scope and potential biases introduced by focusing only on NMP-level perspectives.

4. Limited Cross-Country Comparison

- The manuscript discusses SNT implementation country by country, but lacks an effective cross-country synthesis.
  - What are the key similarities and differences in how each country implemented SNT?
  - Are there country-specific barriers that affected implementation differently (e.g., governance, funding constraints, health system structure)?
  - Suggested Revision: Introduce a comparative analysis table summarizing differences in implementation approach, challenges, and perceived benefits.

5. Impact Assessment of SNT on Malaria Outcomes

- The manuscript reports that SNT led to improved strategic planning, but provides limited evidence of its direct impact on malaria burden reduction.
  - Are there any measurable improvements in malaria incidence, intervention coverage, or funding acquisition attributable to SNT?
  - Suggested Revision: Include specific data (even preliminary) on how SNT changed decision-making and improved malaria program outcomes.

6. Discussion and Recommendations

- The discussion lacks concrete takeaways for malaria program managers and policymakers.
  - What specific lessons can other malaria-endemic countries learn from this study?
  - What are the actionable steps for countries considering SNT implementation?
  - Suggested Addition: Provide a structured set of recommendations on how SNT can be operationalized in different malaria-endemic contexts.

**Minor Comments**

1. Language and Readability

- The manuscript contains excessively long and complex sentences that make it difficult to read.
  - Example:
    - *"Participants expressed that SNT outputs were used to inform national strategic plans and prioritized plans, that the process incentivized improvements in data collection and data quality, and that NMPs were strongly motivated to grow their capacity to conduct more steps of the SNT analysis process internally."*
  - Suggested Revision:
    - *"Participants reported that SNT helped shape national strategic plans, improved data quality, and motivated NMPs to develop internal analytical capacity."*

2. Overuse of Acronyms

- The manuscript frequently uses SNT, NMP, WHO, HBHI, IRS, SMC, ACTs, often without proper reintroduction in later sections.
  - Suggested Fix: Provide a glossary of key terms and acronyms.

3. Abstract Needs Refinement

- The abstract repeats details already present in the main text and could be more concise and impactful.
  - Suggested Fix: Reduce methodological details in the abstract and focus more on key findings and policy implications.

4. Figure and Table Formatting

- Table captions are too generic and do not sufficiently explain what the data represents.
  - Example: Instead of *"Table 1: Summary of SNT implementation steps"*, use *"Table 1: Steps followed in the subnational tailoring (SNT) process across five malaria programs"*
- Figures (especially maps) lack clarity on geographic regions analyzed.
  - Suggested Fix: Provide more precise labels and explanations.

**Final Verdict: Major Revision**

The manuscript is well-structured but lacks analytical depth and clear cross-country comparisons. Key areas requiring revision include improving the conceptual framework, providing stronger evidence of impact, refining the discussion, and making the language more concise. Once these issues are addressed, the manuscript will make a valuable contribution to malaria program planning and implementation research.
