# Supplementary material for: Subnational tailoring of malaria interventions for strategic planning and prioritization: Experience and perspectives of five malaria programs
Source: PLOS Glob Public Health. 2025 May 28;5(5):e0003811. doi: 10.1371/journal.pgph.0003811 (PMC12118815; doi:10.1371/journal.pgph.0003811)
Supplement: Attachment — Submitted filename: reviewer comments.docx [file pgph.0003811.s006.docx]

We thank the reviewers for their truly very thoughtful and insightful comments, which have really improved our paper, and we are grateful that they have found it to be an interesting read.

**Reviewer #1:**

Congratulations on a really interesting and valuable paper.

On my first read I considered if some sections could be reduced since the paper is quite long, however given the limited other literature providing similar feedback on this topic and the comprehensive approach that you have taken, I think the length is justified.

I have no other edits to suggest, and recommend the paper be accepted.

We thank the reviewer for their positive assessment and are glad they found this paper interesting and valuable.

**Reviewer #2:**

The study provides an interesting and useful analysis of the experience of participants in the SNT process, which is highly topical given WHO’s ongoing development of an SNT implementation manual. Of particular interest (to me at least) are the reported challenges relating to subnational partners and the need for improved in-country mathematical modelling expertise.

As a health economist with limited experience in qualitative research methods, I have primarily focused on how the paper describes the SNT process and its application for efficient priority-setting.

MAJOR COMMENTS

High-level comments mostly relate to the framing of the study and the rationale for SNT.

1. The Introduction notes several factors contributing to stalled progress in reducing malaria morbidity and mortality (lines 55-57) but does not clearly explain why, nor really get to the underlying drivers of this stalled progress. For example, “sub-optimal malaria intervention coverage” is mentioned as a factor; this is due to a stagnation in global funding for malaria, and the increasing difficulty (and cost) of expanding coverage to harder-to-reach-populations. The impact of insecticide and antimalarial resistance should also be explicitly mentioned; such resistance has reduced intervention effectiveness and required the adoption of more expensive alternatives.

Thank you for suggesting these additional factors: we have added them to the introduction (L59-61: “Many factors contribute to rising or stable malaria incidence in high-burden countries, including the underlying intensity of malaria transmission, socio-demographic and epidemiologic risk factors, poor access to care, sub-optimal malaria intervention coverage including in hard-to-reach areas, poor implementation quality, rising resistance to insecticides and antimalarials, and stagnation in global funding for malaria”). As far as we are aware, there is no real consensus on which factors are really driving stalled progress, and it may be a combination or setting-specific.

2. The origin of SNT as part of the HBHI approach is described (although this is very lightly referenced, particularly lines 67-107). However, the underlying rationale for SNT is not clearly explained. The effectiveness and cost-effectiveness of interventions varies by geographic area and population, and hence a more efficient allocation of resources is achieved by tailoring intervention delivery based on the stratification of various components of malaria risk. Heterogeneity in malaria risk (which is not mentioned until the Discussion section) underpins the rationale for SNT, and is increasing as incidence declines in many areas. For further detail, refer to (and consider citing) WHO’s recent updates on SNT: ‘Update on subnational tailoring of malaria interventions and strategies’ (Malaria Policy Advisory Group Meeting, 4-5 March 2024) and ‘Guiding principles for prioritizing malaria interventions in resource constrained country contexts to achieve maximum impact’ (May 2024).

We thank the reviewer for pointing out this key omission and have added text on the rationale for subnational tailoring (L91-96: “In SNT, intervention strategies are tailored to individual subnational units, accounting subnational heterogeneity in malaria risk and other local variation in determinants of malaria transmission such as seasonality, vector behavior, and access to health services. SNT moves away from a one-size-fits-all approach to intervention targeting and instead provides a process by which effectiveness and cost-effectiveness of intervention strategies can be optimized under a limited resource envelope.”).

3. The Introduction could also provide a clearer rationale for the importance of the study, including the benefits of an improved understanding of participant experiences of the SNT process.

We have added that the goal of understanding and improving the SNT process is to work toward countries becoming self-reliant in the use of data to inform priority-setting (L147-8: “understanding the NMP experience with the SNT process is necessary to identify successful elements, remaining gaps, and approaches that need adjustment, such that countries become self-reliant in the use of data to inform priority-setting.”).

MINOR COMMENTS

1. Abstract: Consistent with the major comments, the abstract should also provide further background on why SNT is important in order to maximise the health impact of scarce resources across heterogenous areas and populations.

We have added a sentence to the abstract (using the reviewer’s wording, which is really perfect), to explain SNT (L22-24: “SNT is a data-informed method of developing intervention plans that maximize health impact under limited resources across heterogenous areas and populations.”).

2. Line 44: Chemoprevention is mentioned, however this is not supported by the reference provided (Bhatt et al).

We have added a citation to the Access SMC study to support the claim of impact of chemoprevention (L46).

3. Lines 208-9: Were the interviews transcribed and translated by members of the research team?

We have added further detail to the methods section (L220-225): “Interview recordings were transcribed by the transcription company Multilingual Connections. Transcriptions were subsequently quality-assessed against the original audio by the research team (LO and JG). Transcriptions of French interviews were translated into English using the software DeepL. English translations were reviewed next to the original French by bilingual members of the research team (LO, GAO, and JG) to ensure accurate translation transcript content, key acronyms, and malaria terminology.” Translations of French transcripts to English enabled more efficient analysis.

4. Lines 415-32: The analysis relating to “prioritization … to account for costs” is not clearly explained. From the quotes it appears that activities that were prioritized as part of the SNT process were later excluded due to their cost. These raises an important issue regarding the SNT process: prioritisation is currently done based on effectiveness and then subsequently costed, rather than prioritising based on cost-effectiveness – which would take into account heterogeneity of costs and enable more efficient resource allocation.

The reviewer is correct. It would be great to be able to do prioritization using cost-effectiveness estimates. However, at the time it was not possible due to lack of data on costs (including subnational data on costs and implementation costs), but this is a great area for improvement in future rounds of SNT. We have added a note that up until this point, SNT had focused on effectiveness rather than cost-effectiveness (L430-431: “Since SNT until this point had focused on effectiveness rather than cost-effectiveness, in some cases, further prioritization was necessary to account for costs”). While we know anecdotally that this was due to lack of cost data, participants did not mention this, nor did they comment on the non-use of cost-effectiveness, so we have chosen not to elaborate further here.

5. Line 916: This is the first time heterogeneity is mentioned, although it is central to the rationale for SNT. Note that heterogeneity is not just in terms of malaria transmission, but many other factors of malaria risk (including insecticide and drug resistance, access to care, etc).

This is addressed with the explanation of SNT that has been added to the introduction (L91-96). We have also added to this sentence in the discussion that heterogeneity also includes malaria determinants (L932: “which would better represent the heterogeneity of malaria transmission and its determinants”).

6. Line 977: This section could refer to efficiency more explicitly – i.e. improving the health impact of limited malaria control resources.

We have added that NMPs found SNT to be successful for prioritization under resource constraints (L996: “SNT was a highly successful approach for the strategic use of information in decision-making, including for prioritization under resource constraints”). Whether health impact has actually improved remains to be seen!

**Reviewer #3:**

I thought this paper was well written and an excellent complement to published examples of the SNT outputs. However, I do think some of those concrete examples of use of SNT outputs could be reiterated here rather than just referenced. These concrete examples would bolster the positive claims of the qualitative interviews.

Specific comments:

- ln. 57: insecticide resistance was likely a factor here too for both nets and IRS

Thanks for this suggestion, we have added it to the introduction (L60: “Many factors contribute to rising or stable malaria incidence in high-burden countries, including the underlying intensity of malaria transmission, socio-demographic and epidemiologic risk factors, poor access to care, sub-optimal malaria intervention coverage including in hard-to-reach areas, poor implementation quality, rising resistance to insecticides and antimalarials, and stagnation in global funding for malaria”).

- ln 109 and 116: this paragraph might be well illustrated with a table to show countries/types of assistance, etc.

We have added a citation to the meeting notes of the Malaria Policy Advisory Group Meeting of 4-5 March 2024, where there is a great table that summarizes Phase 2 SNT activities (L129).

- ln 134: examples of SNT use. Several of the articles referenced were published too early to have been the products of the SNT process described here (although they were using subnational data to make decisions). However, for a few of the later publications, there are concrete examples of how SNT changed the status quo...it would be very useful to have a few of these concrete examples in this article.

(responded to along with the following comment)

- ln 885-888: can you give concrete examples of decisions made using SNT that might not have happened in the absence of the SNT process. Some of the papers in the references contain actual examples and perhaps some of them could be cited here.

We agree with the reviewer that concrete examples of how decisions made using SNT would be really interesting to consider, and that understanding those decisions would be a key part of evaluating the impact of SNT. However, our study doesn’t seek to associate the participants interviewed with the decisions made, nor to evaluate the quality of those decisions. Rather we seek to understand participants’ experience of the process, which in part includes their perceived impact of SNT, whether or not that reflects the actual impact of SNT. Thus we have presented the decisions that participants themselves chose to highlight (Results sections on Strategic planning and intervention prioritization).

From a broader perspective, in light of the many discussions about measuring the impact of SNT, it is challenging to establish the baseline relative to which impact should be measured. Since SNT is about using data to inform decisions, the counterfactual would be decisions made without considering data, or without considering data using the systematic approach of SNT. The referenced papers discuss how decisions made using SNT were different from status quo interventions, but they don’t address how SNT changed the decision-making process itself, nor do they address the suitability of status quo interventions relative to the epidemiological and resource context of the period during which those intervention strategies were decided. As the impact of SNT on decisions and on malaria is a very complex topic, and our paper is already very long, we have chosen not to go into more detail or discussion on the impact of SNT.

- References: some look like preprints (not peer reviewed). If the paper has subsequently appeared after peer-review, please update the reference

We have checked that the two preprints are still just preprints, but we will check again and update prior to publication of our paper.

**Reviewer #4:**

The manuscript presents a qualitative study exploring the perspectives of National Malaria Programs (NMPs) on the use of Subnational Tailoring (SNT) for malaria strategic planning and prioritisation. While the study is valuable in documenting experiences across five countries, several areas require improvement to enhance clarity, strengthen the analytical depth, and improve the overall presentation.

Major Comments

1. Conceptual Clarity and Rationale for SNT Approach
   a. The manuscript describes SNT as a novel approach but does not clearly differentiate it from existing malaria stratification methods used in endemic countries.
   b. How does SNT differ from traditional malaria stratification approaches used by WHO or NMCPs?
   c. What are the specific advantages of SNT over conventional methods in terms of decision-making?
   d. Was there a preceding malaria stratification framework in these countries before adopting SNT?
   e. Suggested Revision: Provide a clearer theoretical grounding for SNT and its distinct value-add.

We have added text on the rationale for subnational tailoring to the introduction (L91-96: “In SNT, intervention strategies are tailored to individual subnational units, accounting subnational heterogeneity in malaria risk and other local variation in determinants of malaria transmission such as seasonality, vector behavior, and access to health services. SNT moves away from a one-size-fits-all approach to intervention targeting and instead provides a process by which effectiveness and cost-effectiveness of intervention strategies can be optimized under a limited resource envelope.”).

2. Overuse of WHO-Centric Perspective. The WHO's role in SNT implementation is strongly emphasized, but the manuscript lacks a critical discussion on:
   a. How NMPs perceived WHO’s role—was it more of a technical enabler or a decision-making authority?
   b. Did WHO’s involvement introduce limitations, such as prescriptive frameworks that may not fully align with country-specific needs?
   c. Suggested Addition: Provide a balanced discussion on both facilitating and limiting aspects of WHO’s involvement.

Since the objective of the study is to evaluate participant perceptions of the WHO approach, it is necessarily WHO-centric. We include data that speaks to participants’ perception of WHO’s role, which clearly states that they saw WHO as a technical enabler rather than a decision-making authority (L704). We are clear about this limitation of our study to speak to other SNT experiences (L1015-1019).

3. Participant Selection and Generalizability. The study relies on interviews with 12 NMP representatives from five countries. However:
   a. Why were only NMP representatives included? Were perspectives from other stakeholders (e.g., funders, health workers, community leaders) considered?
   b. Suggested Revision: Acknowledge the limited scope and potential biases introduced by focusing only on NMP-level perspectives.

We thank the reviewer for this suggestion and agree that it would be valuable to also gather perspectives from other stakeholders, and together build a holistic view of the experience of SNT. As research into SNT is actively unfolding, we chose to first prioritize the perspectives of NMPs, who are at the center of SNT, and anticipate that future research by us and other groups will eventually enable the systemic understanding that the reviewer recommends. Our exclusive focus on NMP perspectives is indicated in the title and in the framing of all sections, so that readers can clearly understand the scope of this study.

4. Limited Cross-Country Comparison. The manuscript discusses SNT implementation country by country, but lacks an effective cross-country synthesis.
   a. What are the key similarities and differences in how each country implemented SNT?
   b. Are there country-specific barriers that affected implementation differently (e.g., governance, funding constraints, health system structure)?
   c. Suggested Revision: Introduce a comparative analysis table summarizing differences in implementation approach, challenges, and perceived benefits.

While a country-by-country comparison of their implementation of SNT would be fascinating, we feel that is a separate research question from NMPs’ perspectives of the SNT experience, which is our focus in this manuscript. We hope that future research will pursue the reviewer’s suggestion.

5. Impact Assessment of SNT on Malaria Outcomes. The manuscript reports that SNT led to improved strategic planning but provides limited evidence of its direct impact on malaria burden reduction.
   a. Are there any measurable improvements in malaria incidence, intervention coverage, or funding acquisition attributable to SNT?
   b. Suggested Revision: Include specific data (even preliminary) on how SNT changed decision-making and improved malaria program outcomes.

This is a very interesting suggestion from the reviewer and definitely worth further research. While it would add a fascinating dimension, our study is focused on NMPs’ self-reported perspectives, and thus a quantitative epidemiological assessment is out of scope for this work.

6. Discussion and Recommendations. The discussion lacks concrete takeaways for malaria program managers and policymakers.
   a. What specific lessons can other malaria-endemic countries learn from this study?
   b. What are the actionable steps for countries considering SNT implementation?
   c. Suggested Addition: Provide a structured set of recommendations on how SNT can be operationalized in different malaria-endemic contexts.

Global partners and donors are the primary audience for this piece, with NMPs who have not yet implemented SNT a secondary audience. We have made recommendations on capacity development for SNT (L953-958), enabling factors for analysis partners (L981-991), and broader implications for priority-setting (L995-1003). NMPs and their partners may also find interesting takeaways regarding the importance of data and enablers of data use (L918-928). As the focus of our paper is not SNT operationalization nor how countries can implement it, we have not included recommendations or steps toward operationalization.

Minor Comments

Language and Readability: The manuscript contains excessively long and complex sentences that make it difficult to read.

- Example: "Participants expressed that SNT outputs were used to inform national strategic plans and prioritized plans, that the process incentivized improvements in data collection and data quality, and that NMPs were strongly motivated to grow their capacity to conduct more steps of the SNT analysis process internally."
- Suggested Revision: "Participants reported that SNT helped shape national strategic plans, improved data quality, and motivated NMPs to develop internal analytical capacity."

While the reviewer’s suggestion does indeed shorten this sentence, we feel that it loses important detail, specificity, and nuance that are key aspects of our findings.

Overuse of Acronyms: The manuscript frequently uses SNT, NMP, WHO, HBHI, IRS, SMC, ACTs, often without proper reintroduction in later sections.

- Suggested Fix: Provide a glossary of key terms and acronyms.

A list of abbreviations is provided after the Conclusions section.

Abstract Needs Refinement. The abstract repeats details already present in the main text and could be more concise and impactful.

- Suggested Fix: Reduce methodological details in the abstract and focus more on key findings and policy implications.

We are not sure what the reviewer is referring to, as there is one sentence in the abstract (L27-30: “As SNT was a new approach, this study used semi-structured interviews to understand the perspectives and experiences of personnel from five NMPs (Burkina Faso, Ghana, Guinea, Nigeria, and Togo) that undertook SNT between 2019 and 2023.”) that describes methodological details, and key findings and implications are noted in the following 4 sentences (L30-39).

Figure and Table Formatting. Table captions are too generic and do not sufficiently explain what the data represents.

- Example: Instead of "Table 1: Summary of SNT implementation steps", use "Table 1: Steps followed in the subnational tailoring (SNT) process across five malaria programs"
- Figures (especially maps) lack clarity on geographic regions analyzed.
- Suggested Fix: Provide more precise labels and explanations.

We are not sure what the reviewer is referring to, as there are no tables, figures, or maps in our manuscript.

**Final Verdict: Major Revision**

The manuscript is well-structured but lacks analytical depth and clear cross-country comparisons. Key areas requiring revision include improving the conceptual framework, providing stronger evidence of impact, refining the discussion, and making the language more concise. Once these issues are addressed, the manuscript will make a valuable contribution to malaria program planning and implementation research.

See responses above to individual points the reviewer has raised.
